# Fucoidan Protects Against Cadmium-Induced Cytotoxicity in PK-15 Cells by Restoring Autophagic Flux: Involvement of the TFEB Signaling Pathway

**DOI:** 10.3390/toxics14050430

**Published:** 2026-05-13

**Authors:** Haobo Qu, Xiaoyu Zhao, Yifan Wang, Sichao Mao, Xingxiang Chen, Kehe Huang, Xinyi Xu

**Affiliations:** 1 College of Veterinary Medicine, Nanjing Agricultural University, Nanjing 210095, China; quhaobo@stu.njau.edu.cn (H.Q.); xyzhao0404@163.com (X.Z.); wangyifan@stu.njau.edu.cn (Y.W.); scmao@njau.edu.cn (S.M.); cxx@njau.edu.cn (X.C.); khhuang@njau.edu.cn (K.H.); 2 Institute of Animal Nutritional Health, Nanjing Agricultural University, Nanjing 210095, China

**Keywords:** fucoidan, cadmium, autophagy, TFEB

## Abstract

Cadmium (Cd) is a persistent environmental pollutant that poses a significant health risk to humans and animals, with acute exposure known to induce kidney injury. Fucoidan (Fc), a natural bioactive polysaccharide derived from brown algae, exhibits diverse biological activities; however, its potential to protect against Cd-induced kidney damage and the underlying mechanisms remain unclear. In this study, we investigated the effects of Fc on Cd-induced renal injury in vitro and further explored the role of transcription factor EB (TFEB) in regulating autophagy in its protective mechanism. Our results demonstrate that in Cd-exposed porcine kidney cells (PK-15), Fc suppressed the expression of renal inflammatory factors (TNF-α, IL-1β) and kidney injury markers (NGAL, NTN-1, KIM-1), reduced reactive oxygen species (ROS) production, and downregulated apoptosis-related proteins (cleaved caspase-3 and cleaved caspase-9). Mechanistically, Fc upregulated TFEB protein expression, enhanced the levels of lysosomal function-related proteins (Cathepsin B, CTSB; Cathepsin D, CTSD), and reversed Cd-induced autophagic flux blockade. Importantly, TFEB silencing abolished the protective effects of Fc. Collectively, these findings suggest that Fc exerts renoprotective effects against Cd-induced injury by restoring autophagic flux, a process that involves TFEB.

## 1. Introduction

Cadmium is a ubiquitous environmental toxicant [1,2] that accumulates in mammals primarily via industrial pollution and contaminated food chain [2,3,4], leading to multi-organ toxicity [5]. Among affected organs, the kidney is the principal target [6] and vulnerable to injury. This damage appears as impaired tubular function, reduced glomerular filtration, and progressive fibrosis [7,8]. Long-term Cd exposure markedly increases the risk of developing chronic kidney disease [9] and ultimately precipitates end-stage renal failure [10].

Autophagy is indispensable for the maintenance of renal homeostasis [11]. Conditional deletion of core autophagy genes (e.g., ATG5 or Six2-Cre deletion [12]) precipitates overt renal injury. Long-term Cd exposure disrupts this protective process by inducing lysosomal alkalinization, which inactivates key hydrolytic enzymes (e.g., cathepsins) and impairs autophagosome–lysosome fusion, thereby blocking autophagic flux [13]. This disruption leads to lysosomal dysfunction and disorganization of the autophagic pathway, ultimately resulting in cellular damage and contributing to Cd-induced nephrotoxicity [14].

Fucoidan, a sulphated polysaccharide extracted from brown algae, exhibits robust antioxidant, anti-inflammatory, and immunoregulatory bioactivities [15,16,17]. Accumulating evidence indicates that it confers renoprotection [18,19,20]. Although research data remain limited, recent studies have shown that Fc ameliorates cisplatin-induced acute kidney injury [21], and attenuates hyperuricemic nephropathy via inhibition of inflammation and apoptosis [22]. Fc has been shown to enhance autophagy across multiple biological systems, contributing to its protective effects in various disease models. For instance, Fc alleviates dextran sulfate sodium-induced ulcerative colitis by promoting autophagy and modulating the intestinal microbiota [23]. Furthermore, studies demonstrate that Fc can regulate JAK2/STAT3-mediated autophagy and apoptosis, thereby attenuating doxorubicin-induced acute cardiotoxicity [24].

TFEB, a key transcriptional regulator of autophagy–lysosomal function [25], is suppressed by Cd, resulting in disrupted autophagic flux and disruption of cellular homeostasis [26]. Activation of TFEB is therefore viewed as a promising therapeutic strategy. Although Fc has been shown to ameliorate atherosclerosis [26] and cisplatin-induced kidney injury [27] by enhancing TFEB, its ability to activate TFEB and thus protect against Cd-induced renal damage remains unexplored.

This study aimed to determine the renoprotective efficacy of Fc against Cd nephrotoxicity and to explore whether this protection involves TFEB in the restoration of autophagic flux.

## 2. Materials and Methods

### 2.1. Materials

Fucoidan (Fc, purity > 95%, Mw = 8.23 kDa, P572406004) was purchased from Mingyue Seaweed Group Co., Ltd. (Qingdao, China). Cadmium chloride (CdCl_2_, purity > 95%, C116344-10g) was purchased from Aladdin (Shanghai, China). DAPI (ID2250-5mg) and chloroquine (CQ, IC44409-1ml) were purchased from Solarbio (Beijing, China). The cell counting kit 8 (CCK8, CK04) assay was purchased from Dojindo (Kumamoto, Japan). Antibodies for GAPDH (60004-1-Ig), p62 (18420-1-AP) and LC3B (14600-1-AP) were purchased from Proteintech (Wuhan, China); caspase-3 (A19664), caspase-9 (A2636), CTSB (A0967), CTSD (A19680) and TFEB (A21657) were purchased from ABclonal (Wuhan, China). RIPA lysis buffer (RM02998) was also obtained from ABclonal. Trizol reagent (AG21101) M-MLV reverse transcriptase (AG11754) and SYBR green real-time PCR kit (AG11761) were purchased from (Accurate Biotechnology (Changsha, China). PK-15 cells, a porcine kidney epithelial cell line, were selected as a well-established model for studying heavy metal-induced nephrotoxicity and autophagic cytoprotection, and were obtained from the Institute of Biochemistry and Cell Biology (Shanghai, China). For cell transfection, small interfering RNA against TFEB (siTFEB) (sense: GAC-GAAGGUUCAACAUCAATT; antisense: UUGAU-GUUGAACCUUCGUCTT) or negative control RNAi (siNC) were purchased from Shenggong (Shanghai, China) and the green fluorescent protein- LC3 (GFP-LC3) plasmid was obtained from Jiman Biotechnology Co., Ltd. (Shanghai, China).

### 2.2. Cell Culture and Treatment

PK-15 cells were cultured in Dulbecco’s Modified Eagle Medium (DMEM, Gibco, Grand Island, NY, USA) containing 10% fetal bovine serum, 1% penicillin–streptomycin, at 37 °C under 5% CO_2_ in a humidified incubator.

Cells were pretreated with Fc (30, 60, or 120 μg/mL; dissolved in PBS) for 12 h. After washing twice with PBS, the cells were then exposed to CdCl_2_ (7.5 μM; dissolved in sterile deionized water) for 24 h. For experiments designed to inhibit autophagy, chloroquine (CQ; 40 μM; dissolved in dimethyl sulfoxide, DMSO) was added during the final 4 h of the CdCl_2_ treatment period [28]. The final concentration of DMSO in the culture medium did not exceed 0.1% (*v*/*v*).

### 2.3. Cell Viability and Apoptosis Assay

The cell viability of PK-15 cells was assessed using the Cell Counting Kit-8 (CCK-8) assay [29]. Following the indicated treatments, 10 µL of CCK-8 solution was added to each well containing 100 µL of culture medium. After incubation at 37 °C for 1.5 h, the absorbance at 450 nm was measured using a microplate reader.

### 2.4. Detection of Intracellular Reactive Oxygen Species (ROS)

Intracellular ROS levels were measured using a DCFH-DA fluorescent probe. PK-15 cells were seeded in 12-well plates at a density of 8 × 10^4^ cells per well and allowed to reach 50–70% confluence before drug treatment for 24 h. After washing twice with pre-warmed PBS, cells were incubated with 20 μM DCFH-DA in serum-free DMEM at 37 °C for 30 min in the dark. Following three washes with PBS, fluorescence images were immediately captured using an inverted fluorescence microscope at an excitation wavelength of 525 nm.

### 2.5. Laser Confocal Microscopy

PK-15 cells were transfected with GFP-LC3 plasmid using jetPRIME reagent according to the manufacturer’s instructions. After 12 h, cells were subjected to the indicated experimental treatments [30]. Following treatment, cells were washed with PBS and mounted with antifade mounting medium containing DAPI. Fluorescent images were acquired using a ZEISS confocal laser scanning microscope under consistent scanning parameters, including laser power, gain, and pinhole size, to ensure comparability across all samples.

### 2.6. Quantitative Real-Time PCR (qRT-PCR) Analysis

Total RNA was extracted using Trizol reagent and cDNA was synthesis with M-MLV reverse transcriptase. Quantitative real-time PCR (qPCR) was performed using SYBR Green dye on a ABI Prism Step One Plus Real-Time PCR System. Primer sequences are listed in Table S1, and data were calculated according to ΔΔCt method [31].

### 2.7. Western Blot

Total protein was extracted from PK-15 cells using RIPA lysis buffer containing 1% protease inhibitor cocktail, as previously described [32]. Target protein abundance was normalized to GAPDH (loading control) and quantified by densitometry using ImageJ software (v1.54g).

### 2.8. Statistical Analysis

Data are presented as mean ± SD from ≥ 3 independent experiments. Statistical analyses were performed with *t*-test or one-way analysis of variance (ANOVA) followed by Tukey’s test. *p* value < 0.05 was considered statistically significant and *p* < 0.01 highly significant. All graphs were generated using GraphPad Prism 8 software.

## 3. Results

### 3.1. Fucoidan Attenuated Cadmium-Induced Cellular Damage in PK-15 Cells

**Figure 1:**
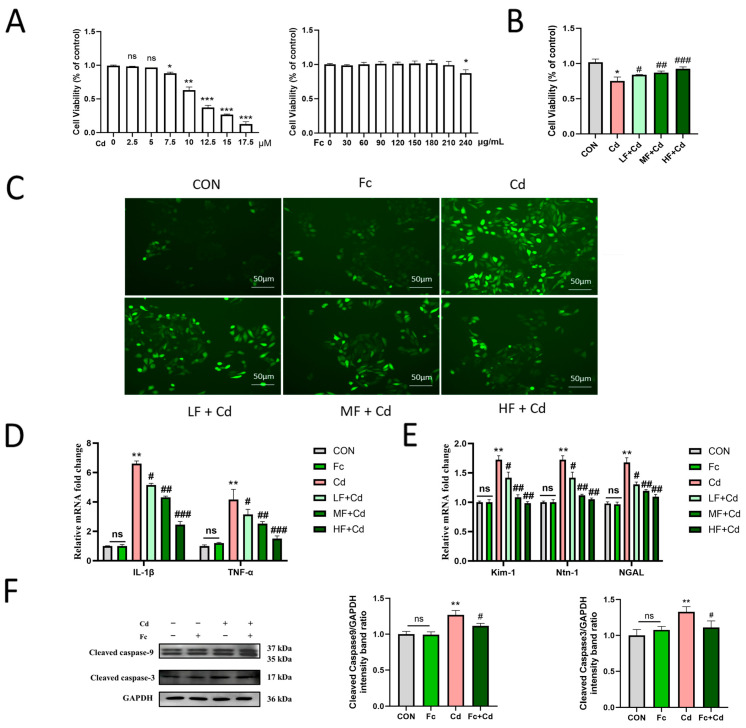
Effects of Fc on Cd-induced oxidative stress, inflammation, and apoptosis in PK-15 cells. (**A**) Effects of combined treatment with Cd and Fc on PK-15 cell viability. (**B**) Cell viability measured by CCK-8 assay after treatment with Cd or Fc. (**C**) Intracellular ROS levels visualized by fluorescence microscopy (scale bar: 50 μm). (**D** and **E**) mRNA expression of NGAL, Ntn-1, KIM-1, IL-1β, and TNF-α in PK-15 cells determined by RT-PCR. (**F**) Representative protein expression analyzed by Western blot. Data are expressed as mean ± SEM from three independent experiments (*n* = 3). * *p* < 0.05, ** *p* < 0.01, *** *p* < 0.001 for Control vs. Cd; # *p* < 0.05, ## *p* < 0.01, ### *p* < 0.001 for  Cd vs. LF/MF/HF + Cd (or Cd vs. Fc + Cd); ns, not statistically significant.

To determine an appropriate challenge dose for subsequent experiments, the cell viability across a range of Cd concentrations was assessed. A total of 7.5 μM Cd was selected as the challenge dose, as it reduced cell viability to approximately 75% of the control (IC25), thereby inducing moderate, non-lethal cytotoxicity that is optimal for assessing protective effects. For Fc, preliminary experiments showed no cytotoxicity at concentrations below 240 μg/mL (Figure 1A). Furthermore, CCK-8 assays revealed that 30, 60, and 120 μg/mL Fc significantly improved cell viability in a dose-dependent manner in Cd-treated cells (Figure 1B). Accordingly, 30, 60, and 120 μg/mL Fc were selected as the low-, medium-, and high-dose protective groups (LF + Cd, MF + Cd, HF + Cd). A control group treated with 120 μg/mL Fc alone was also included to rule out potential cytotoxicity, and no obvious cytotoxicity was observed.

Then, PK-15 cells were pretreated with Fc before Cd exposure to investigate its protective potential. The results showed that Fc suppressed transcriptional upregulation of inflammatory factors Interleukin-1 Beta and Tumor Necrosis Factor-Alpha (IL-1β and TNF-α, Figure 1D) and attenuated expression of renal injury biomarkers KIM-1, Ntn-1 and NGAL (Figure 1E) due to Cd exposure. Concurrently, Fc treatment visibly reduced intracellular reactive oxygen species (ROS) accumulation compared with the Cd-exposed group, as observed by qualitative fluorescence imaging (Figure 1C). Among these concentrations, 120 μg/mL Fc exhibited the most pronounced protective effects in CCK-8 assay, ROS imaging, inflammatory factor detection, and renal injury marker analysis. Therefore, 120 μg/mL Fc was used as the optimal concentration in subsequent experiments. As shown in Figure 1F, Fc markedly reduced apoptosis, as demonstrated by decreased protein levels of the proapoptotic markers cleaved caspase-3 and cleaved caspase-9. Taken together, these data suggest that Fc provides protection against Cd-induced cytotoxicity in PK-15 cells.

### 3.2. Fucoidan Enhances Autophagy in Cadmium-Exposed PK-15 Cells

**Figure 2:**
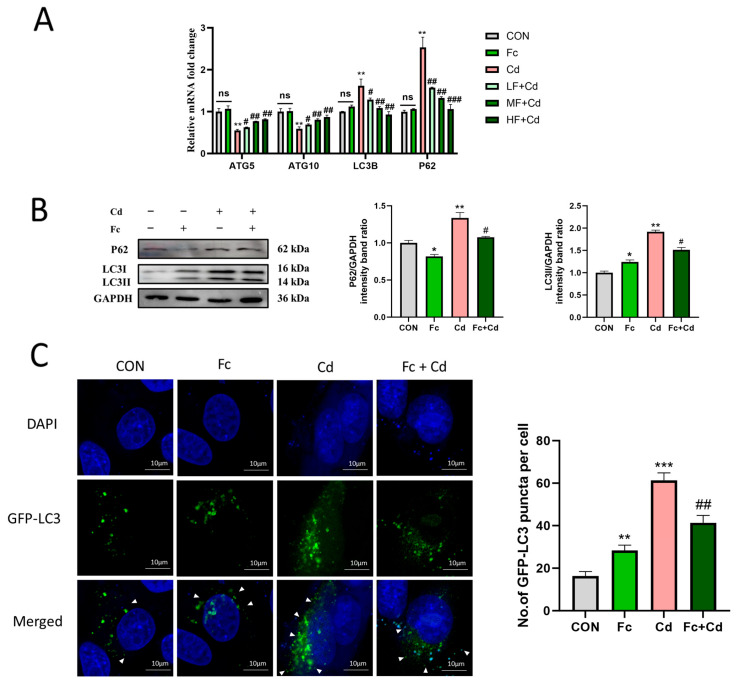
Effect of Fc on autophagy-related markers in Cd-exposed PK-15 cells. (**A**) mRNA expression of ATG5, ATG10, LC3B, and p62 in PK-15 cells. (**B**) Protein levels of p62 and LC3II in PK-15 cells. (**C**) Representative images of GFP-LC3 puncta (green) in transfected PK-15 cells counterstained with DAPI (blue) and observed by laser confocal microscopy (scale bar: 50 μm). White triangles indicate the punctate aggregation of GFP-LC3. Data are expressed as mean ± SEM from three independent experiments (*n* = 3). * *p* < 0.05, ** *p* < 0.01, *** *p* < 0.001 for Control vs. Cd (or Control vs. Fc); # *p* < 0.05, ## *p* < 0.01 for Cd vs. LF/MF + Cd (or Cd vs. Fc + Cd); ### *p* < 0.001 for Cd vs. HF + Cd; ns, not statistically significant.

In Cd-exposed PK-15 cells, dysregulated autophagy was observed, characterized by the accumulation of autophagic substrates LC3II and p62, indicating a potential blockade of autophagic flux. This accumulation was effectively reversed by Fc intervention (Figure 2A,B). Furthermore, the Cd-induced downregulation of key autophagy-related genes, ATG5 and ATG10, was also significantly restored following Fc treatment (Figure 2A). To further verify autophagic flux, quantitative analysis of GFP-LC3 puncta was performed. The number of GFP-LC3 puncta per cell was significantly increased upon Cd exposure, reflecting excessive autophagosome accumulation. In contrast, Fc treatment notably reduced the number of GFP-LC3 puncta (Figure 2C), consistent with relieved autophagic blockade. Collectively, these results suggest that Fc ameliorates Cd-triggered autophagic dysfunction in PK-15 cells and relieves Cd-induced autophagic flux blockade.

### 3.3. Fucoidan Protects Against Cadmium Toxicity in PK-15 Cells by Enhancing Autophagic Flux and Preserving Lysosomal Function

**Figure 3:**
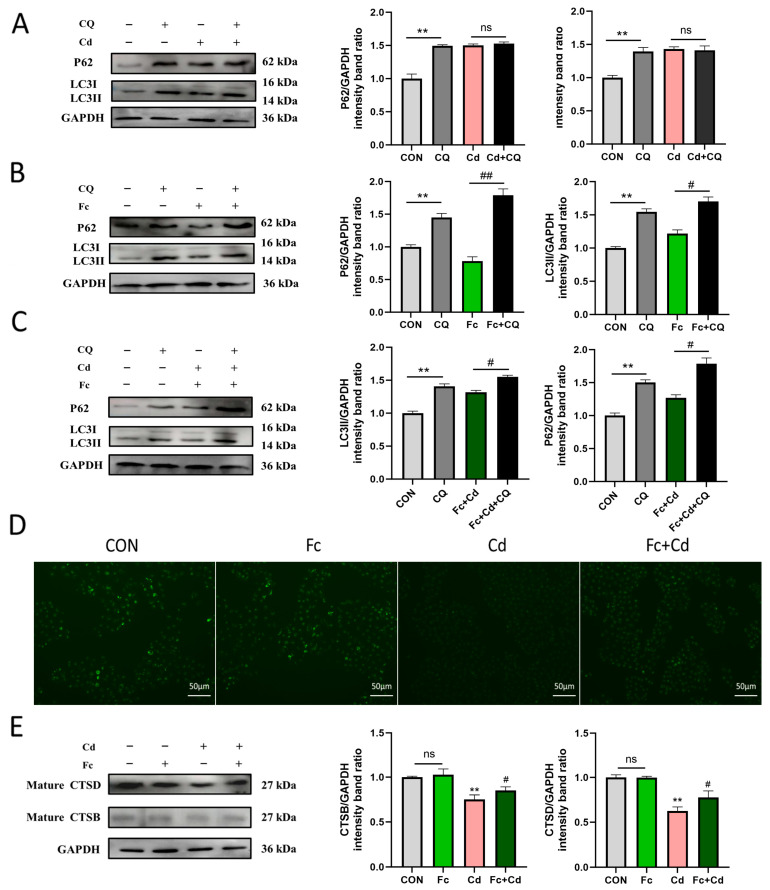
Effect of Fc on the autophagy–lysosome pathway function in Cd-treated PK-15 cells. (**A**–**C**) Protein expression levels of p62 and LC3II in PK-15 cells under indicated treatments, with or without 50 μM chloroquine (CQ), assessed by Western blot. (**D**) Lysosomal abundance visualized by LysoTracker Green staining and observed under a fluorescence microscope (scale bar: 50 μm). (**E**) Protein levels of mature CTSB and mature CTSD in PK-15 cells determined by Western blot. Data are expressed as mean ± SEM from three independent experiments (*n* = 3). For panels (**A**–**D**): ** *p* < 0.01 for Control vs. CQ; # *p* < 0.05, ## *p* < 0.01 for comparisons between groups with and without CQ under the same treatment condition (e.g., Cd vs. Cd + CQ; Fc vs. Fc + CQ; Cd + Fc vs. Cd + Fc + CQ). For panel (**E**): ** *p* < 0.01 for Cd vs. Control; # *p* < 0.05 for Cd vs. Fc + Cd; ns, not statistically significant.

CQ was used to inhibit lysosomal activity. A non-cytotoxic concentration of CQ was selected based on CCK-8 assay (Figure S1). Autophagic flux was then assessed in PK-15 cells. Compared with the corresponding CQ-free groups, CQ co-treatment did not further increase LC3II or p62 levels in Cd-treated cells (Figure 3A). In contrast, CQ addition increased LC3II and p62 levels in Fc-treated cells (Figure 3B). In cells treated with both Cd and Fc, CQ co-treatment also elevated LC3II and p62 levels (Figure 3C). These results indicate that Cd blocks autophagic flux, while Fc restores autophagic degradation capacity in Cd-exposed cells.

Lysosomal dysfunction contributes to autophagic flux blockade. CTSB and CTSD are vital proteases for lysosomal activity. LysoTracker Green staining was used to evaluate lysosomal status. As shown in Figure 3D, Cd weakened lysosomal fluorescence, whereas Fc treatment visibly increased fluorescence intensity. Western blot analysis revealed that Fc treatment significantly restored the protein levels of mature CTSB and CTSD compared with the Cd group (Figure 3E). Together, these data suggest that Fc mitigates Cd-induced lysosomal dysfunction and may contribute to rescuing autophagic flux.

### 3.4. Fucoidan Restores Autophagic Flux and Protects Against Cadmium Toxicity: Involvement of TFEB

**Figure 4:**
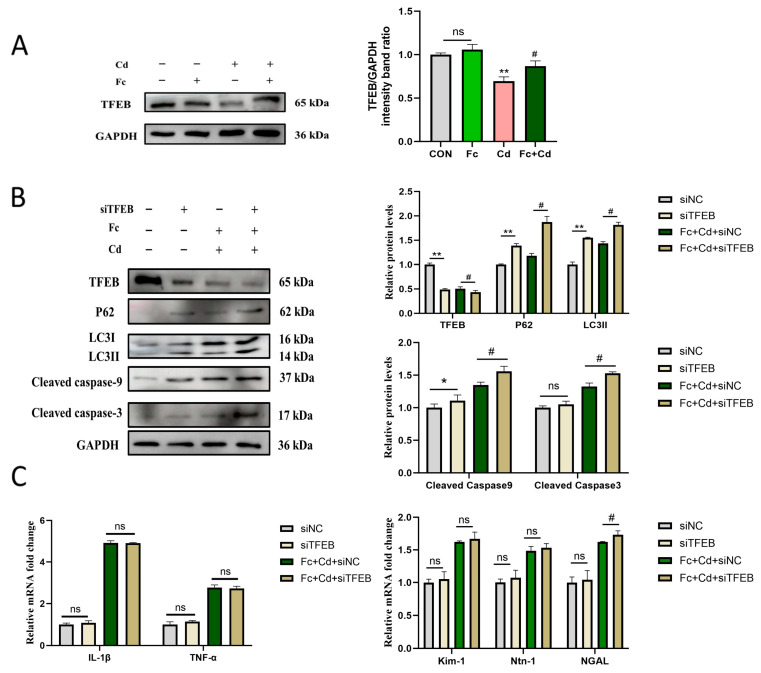
Effect of Fc on TFEB-mediated protection in Cd-treated PK-15 cells. (**A**) Protein expression of TFEB in PK-15 cells measured by Western blot. (**B**) Protein levels of TFEB, p62, LC3II, cleaved caspase-9, and cleaved caspase-3 in transfected cells under indicated treatments. (**C**) mRNA expression of IL-1β, TNF-α, NGAL, KIM-1, and Ntn-1 detected by RT-PCR. Data are expressed as mean ± SEM from three independent experiments (*n* = 3). * *p* < 0.05, ** *p* < 0.01 for CON + siNC vs. CON + siTFEB (or Control vs. Cd); # *p* < 0.05 for Cd + Fc + siNC vs. Cd + Fc + siTFEB (or Cd vs. Fc + Cd); ns, not statistically significant.

TFEB, a master regulator of autophagy and lysosome biogenesis, was downregulated at the protein level by Cd exposure and restored by Fc treatment (Figure 4A). TFEB knockdown reversed the protective effects of Fc, including the reductions in apoptotic markers (cleaved caspase-3 and cleaved caspase-9), normalization of autophagic flux markers (LC3II and p62), and suppression of pro-inflammatory cytokines (IL-1β, TNF-α) and renal injury markers (KIM-1, NGAL, Ntn-1) (Figure 4B,C). These findings suggest that Fc alleviates Cd-induced cellular damage primarily by restoring autophagic flux, a process that involves TFEB.

## 4. Discussion

Cd exerts well-characterized toxic effects on renal cells, and the underlying molecular mechanisms have been extensively studied. Existing evidence indicates that Cd primarily induces persistent damage to renal structure and function through multiple pathways, including the induction of oxidative stress, the triggering of inflammatory responses, and the mediation of apoptosis, ultimately leading to renal dysfunction and tissue pathology [33,34]. As the primary target organ for Cd accumulation and toxicity, the kidneys are susceptible to pathological changes such as renal tubular epithelial cell injury and renal interstitial fibrosis following long-term or low-dose Cd exposure [35], which severely disrupts normal renal physiological homeostasis.

In this study, PK-15 cells were selected as the research model. To establish a moderate cytotoxicity model for evaluating the protective effect of Fc, a Cd concentration that reduced cell viability to approximately 75% was employed. This concentration (7.5 μM) corresponds to the IC25 value, representing a mild but consistent inhibitory effect that is suitable for assessing cytoprotection without causing severe cell death. A previous study using human renal proximal tubule epithelial HK-2 cells reported that treatment with 10 μM Cd for 24 h achieved the IC25 level [36]. This slight difference in concentration may result from variations in species, cellular characteristics, and metabolic profiles, consistent with the differing sensitivity of cell lines to toxicants.

In the present study, Cd exposure induced excessive ROS generation, disrupted redox balance, and triggered oxidative stress in PK-15 cells. Meanwhile, Cd upregulated the expression of inflammatory factors (TNF-α, IL-1β) and kidney injury markers, activating inflammatory responses. Furthermore, Cd significantly promoted apoptosis via the intrinsic apoptotic pathway. These cytotoxic effects are consistent with previous reports [37,38]. Fc is a natural algal polysaccharide with antioxidant, anti-inflammatory, and anti-apoptotic properties [39,40,41]. Our results showed that Fc effectively alleviated Cd-induced cellular damage, as evidenced by suppressed ROS overproduction, reduced inflammation, and inhibited apoptosis. These protective effects are consistent with previous studies in other cell models [42,43,44].

Impaired autophagy is a core mechanism underlying Cd-induced renal cell injury, which was the key regulatory axis investigated in this study. Current research on the relationship between kidney injury and autophagy is extensive, with a general consensus that autophagy is a core physiological process for maintaining renal homeostasis, crucial for metabolic balance, injury repair, and functional maintenance of renal tubular epithelial cells. Dysregulation of the autophagic pathway directly contributes to the onset and progression of various kidney diseases [45,46].

Numerous studies have confirmed that impaired autophagic flux is a key mechanism in Cd-induced renal injury. Cd disrupts lysosomal acidification and reduces the activity of lysosomal hydrolases, thereby inhibiting the fusion of autophagosomes with lysosomes and blocking autophagic degradation [47]. This leads to the accumulation of damaged proteins and organelles, aggravating oxidative stress, inflammation, and apoptosis, and ultimately causing renal cell damage [48,49]. Our results are consistent with this mechanism: 24-h Cd exposure significantly obstructed autophagic flux in PK-15 cells, as evidenced by marked upregulation of the autophagy marker LC3II and substrate protein p62, suggesting increased autophagosome formation but impaired degradation. CQ intervention did not further enhance LC3II and p62 accumulation, which is consistent with the notion that Cd blocks the downstream degradation stage of autophagic flux rather than merely promoting autophagosome formation. Concurrently, Cd exposure induced abnormal lysosomal pH elevation (lysosomal alkalinization) and significantly reduced protein expression of lysosomal cathepsins CTSB and CTSD. These observations further suggest that Cd impairs autophagic flux via lysosomal dysfunction and reduced enzyme activity, consistent with previous reports.

Fc intervention significantly reversed Cd-induced autophagic blockade and effectively restored normal autophagic flux in PK-15 cells. Specifically, Fc treatment markedly reduced the abnormally high expression of LC3II and p62 proteins, indicating restored autophagosome degradation. This regulatory effect is consistent with the autophagy-activating properties of Fc reported in other cellular models. Previous studies have shown that Fc alleviates LPS-induced inflammation by activating autophagy in M2 macrophages and enhances autophagic flux while reducing intracellular lipid accumulation in foam cells, supporting its stable autophagy-regulating activity [50]. In this study, Fc not only restored autophagic function in Cd-injured cells but also partially normalized lysosomal pH, promoted the recovery of CTSB and CTSD, and repaired lysosomal degradation capacity, thereby targeting the upstream cause of autophagic flux blockade. Notably, Fc also promoted physiological autophagy: in normal PK-15 cells, Fc alone upregulated LC3II and downregulated p62, indicating enhanced basal autophagic flux. After chloroquine treatment, the accumulation of LC3II and p62 was significantly higher in the Fc-only group than in the chloro-quine-only control group, suggesting a stronger response to chloroquine. Taken together, these results suggest that Fc enhances basal autophagic flux in a physiologically safe manner without inducing excessive autophagic cell death.

TFEB is a core regulator of cellular homeostasis, autophagy, and lysosomal function, acting as a key hub connecting the autophagy–lysosome system. Numerous studies have confirmed that TFEB coordinately regulates autophagy-related and lysosomal biogenesis genes, thereby activating the autophagy–lysosome pathway to maintain cellular metabolism and stress tolerance [51,52]. Existing evidence indicates that inhibition of TFEB expression and nuclear translocation is a critical molecular feature of Cd-induced kidney injury. Cd im-pairs TFEB activity, aggravating lysosomal dysfunction and autophagic blockade to amplify renal cytotoxicity [53,54]. Based on these findings, our study further suggests that the renoprotective effect of Fc against Cd toxicity involves TFEB. Fc modulates the autophagy–lysosome system with the involvement of TFEB, thereby reversing Cd-induced renal cell injury. Currently, research on Fc-mediated TFEB regulation remains limited, with only one study reporting that Fc attenuates atherosclerosis by activating TFEB [55]. The present study extends this mechanism to Cd-induced kidney injury for the first time, suggesting that TFEB activation may play an important role in the renoprotective effects of Fc. These findings provide a potential therapeutic target for Cd nephrotoxicity and help refine the molecular network of Fc-mediated organ protection, laying a theoretical foundation for future in vivo studies and clinical translation.

Nevertheless, our study has certain limitations. First, the dose-dependency of Fc’s autophagy-enhancing effect was not systematically examined. Second, TFEB activity is regulated by multiple factors, including subcellular localization and post-translational modifications [56,57,58], which were not comprehensively addressed. In addition, the present study only focused on the total protein expression level of TFEB; further detection of TFEB nuclear translocation and its classical downstream targets (such as LAMP1, MCOLN1, and other CLEAR network-regulated lysosomal genes) would be needed to more fully characterize its transcriptional activity in future investigations. Third, autophagic flux was evaluated via indirect markers, and ROS as well as lysosomal status were assessed by qualitative fluorescence imaging. Though widely used, these indirect or semi-quantitative approaches could be further validated with more direct quantitative methods in subsequent studies. Fourth, all experiments were performed in vitro in PK-15 cells, and the protective effects of Fc against Cd-induced renal injury still require in vivo verification. Further animal studies are needed to confirm the translational value and therapeutic potential of Fc.

## 5. Conclusions

In summary, these findings suggest that Fc alleviates Cd-induced renal injury with the involvement of TFEB in restoring autophagic flux. Mechanistically, Fc suppresses Cd-triggered oxidative stress, inflammation, and apoptosis in a manner that involves TFEB; TFEB knockdown abolished the protective effects of Fc. Notably, this study is among the first to link TFEB-associated autophagic flux restoration to the protective mechanism of Fc against Cd-induced renal cell damage, pointing to a potential molecular target for mitigating heavy metal nephrotoxicity.

## Data Availability

The original contributions presented in this study are included in the article/Supplementary Material. Further inquiries can be directed to the corresponding author.

## Supplementary Materials

The following supporting information can be downloaded at: https://www.mdpi.com/article/10.3390/toxics14050430/s1. Figure S1. Effect of different concentrations of chloroquine (CQ) on cell viability after 4 h treatment. Cells were treated with CQ at concentrations of 0, 10, 20, 30, 40, 50, and 60 μM for 4 h, and cell viability was measured by CCK-8 assay and expressed as a percentage of the control (0 μM) group. Data are presented as mean ± SD (*n* = 3). ns, not significant (one-way ANOVA followed by Tukey’s post hoc test). Table S1 The primer sequences for each gene.

## Abbreviations

The following abbreviations are used in this manuscript:

Fc	Fucoidan
Cd	Cadmium
TFEB	Transcription Factor EB
CQ	Chloroquine
ROS	Reactive Oxygen Species
IL-1β	Interleukin-1 Beta
TNF-α	Tumor Necrosis Factor-Alpha
CTSB	Cathepsin B
CTSD	Cathepsin D

## References

[1] Carocci A., Rovito N., Sinicropi M.S., Genchi G. (2014). Mercury toxicity and neurodegenerative effects. Rev. Environ. Contam. Toxicol..

[2] Rafati Rahimzadeh M., Rafati Rahimzadeh M., Kazemi S., Moghadamnia A.A. (2017). Cadmium toxicity and treatment: An update. Casp. J. Intern. Med..

[3] Genchi G., Carocci A., Lauria G., Sinicropi M.S., Catalano A. (2020). Nickel: Human Health and Environmental Toxicology. Int. J. Environ. Res. Public Health.

[4] Liao W., Huang Y., Zhong S., Zhang L., Yu K., Yu S., Su P., Jin C., Yang L., Li F. (2024). Cadmium uptake and transport in vegetables near a zinc-lead mine: Novel insights from Cd isotope fractionation. J. Hazard. Mater..

[5] Tinkov A.A., Gritsenko V.A., Skalnaya M.G., Cherkasov S.V., Aaseth J., Skalny A.V. (2018). Gut as a target for cadmium toxicity. Environ. Pollut..

[6] Satarug S. (2018). Dietary Cadmium Intake and Its Effects on Kidneys. Toxics.

[7] Barregard L., Sallsten G., Lundh T., Mölne J. (2022). Low-level exposure to lead, cadmium and mercury, and histopathological findings in kidney biopsies. Environ. Res..

[8] Holterman W.F., de Voogt P., Peereboom-Stegeman J.H. (1984). Cadmium/zinc relationships in kidney cortex and metallothionein of horse and red deer: Histopathological observations on horse kidneys. Environ. Res..

[9] Cui J., Liu Y., Hao Z., Liu Y., Qiu M., Kang L., Teng X., Tang Y. (2023). Cadmium induced time-dependent kidney injury in common carp via mitochondrial pathway: Impaired mitochondrial energy metabolism and mitochondrion-dependent apoptosis. Aquat. Toxicol..

[10] Bautista C.J., Arango N., Plata C., Mitre-Aguilar I.B., Trujillo J., Ramírez V. (2024). Mechanism of cadmium-induced nephrotoxicity. Toxicology.

[11] Tang C., Livingston M.J., Liu Z., Dong Z. (2020). Autophagy in kidney homeostasis and disease. Nat. Rev. Nephrol..

[12] Kawakami T., Gomez I.G., Ren S., Hudkins K., Roach A., Alpers C.E., Shankland S.J., D’Agati V.D., Duffield J.S. (2015). Deficient Autophagy Results in Mitochondrial Dysfunction and FSGS. J. Am. Soc. Nephrol..

[13] Shao Y., Zheng L., Jiang Y. (2024). Cadmium toxicity and autophagy: A review. Biometals.

[14] Tuffour A., Adebayiga Kosiba A., Addai Peprah F., Gu J., Zhou Y., Shi H. (2023). Cadmium-induced stress: A close look at the relationship between autophagy and apoptosis. Toxicol. Sci..

[15] Li R., Mou J., Zhao L., Hu M., Wang B., Sun Y., Liu J., Qi X., Yang J. (2024). Fucoidan from *Stichopus chloronotus* relieved DSS induced ulcerative colitis through inhibiting intestinal barrier disruption and oxidative stress. Int. J. Biol. Macromol..

[16] Selim H.M., Negm W.A., Hawwal M.F., Hussein I.A., Elekhnawy E., Ulber R., Zayed A. (2023). Fucoidan mitigates gastric ulcer injury through managing inflammation, oxidative stress, and NLRP3-mediated pyroptosis. Int. Immunopharmacol..

[17] Zhang W., Oda T., Yu Q., Jin J.O. (2015). Fucoidan from *Macrocystis pyrifera* has powerful immune-modulatory effects compared to three other fucoidans. Mar. Drugs.

[18] Gao X., Yin Y., Liu S., Dong K., Wang J., Guo C. (2023). Fucoidan-proanthocyanidins nanoparticles protect against cisplatin-induced acute kidney injury by activating mitophagy and inhibiting mtDNA-cGAS/STING signaling pathway. Int. J. Biol. Macromol..

[19] Shi J., Xu Y., Zhang K., Liu Y., Zhang N., Zhang Y., Zhang H., Liang X., Xue M. (2025). Fucoidan Oligosaccharide Supplementation Relieved Kidney Injury and Modulated Intestinal Homeostasis in D-Galactose-Exposed Rats. Nutrients.

[20] Wang M.Z., Wang J., Cao D.W., Tu Y., Liu B.H., Yuan C.C., Li H., Fang Q.J., Chen J.X., Fu Y. (2022). Fucoidan Alleviates Renal Fibrosis in Diabetic Kidney Disease via Inhibition of NLRP3 Inflammasome-Mediated Podocyte Pyroptosis. Front. Pharmacol..

[21] Gao X., Wang J., Wang Y., Liu S., Dong K., Wu J., Wu X., Shi D., Wang F., Guo C. (2022). Fucoidan-ferulic acid nanoparticles alleviate cisplatin-induced acute kidney injury by inhibiting the cGAS-STING pathway. Int. J. Biol. Macromol..

[22] Jiang T., Zhu F., Gao X., Wu X., Zhu W., Guo C. (2025). Naringenin loaded fucoidan/polyvinylpyrrolidone nanoparticles protect against folic acid induced acute kidney injury in vitro and in vivo. Colloids Surf. B Biointerfaces.

[23] Li S., Qian Q., Yang H., Wu Z., Xie Y., Yin Y., Cui Y., Li X. (2024). Fucoidan alleviated dextran sulfate sodium-induced ulcerative colitis with improved intestinal barrier, reshaped gut microbiota composition, and promoted autophagy in male C57BL/6 mice. Nutr. Res..

[24] Zhang J., Sun Z., Lin N., Lu W., Huang X., Weng J., Sun S., Zhang C., Yang Q., Zhou G. (2020). Fucoidan from Fucus vesiculosus attenuates doxorubicin-induced acute cardiotoxicity by regulating JAK2/STAT3-mediated apoptosis and autophagy. Biomed. Pharmacother..

[25] Huang Y., Luo G., Peng K., Song Y., Wang Y., Zhang H., Li J., Qiu X., Pu M., Liu X. (2024). Lactylation stabilizes TFEB to elevate autophagy and lysosomal activity. J. Cell Biol..

[26] Zhao Y., Li Z.F., Zhang D., Wang Z.Y., Wang L. (2021). Quercetin alleviates Cadmium-induced autophagy inhibition via TFEB-dependent lysosomal restoration in primary proximal tubular cells. Ecotoxicol. Environ. Saf..

[27] Zhu L., Yuan Y., Yuan L., Li L., Liu F., Liu J., Chen Y., Lu Y., Cheng J. (2020). Activation of TFEB-mediated autophagy by trehalose attenuates mitochondrial dysfunction in cisplatin-induced acute kidney injury. Theranostics.

[28] Klionsky D.J., Abdel-Aziz A.K., Abdelfatah S., Abdellatif M., Abdoli A., Abel S., Abeliovich H., Abildgaard M.H., Abudu Y.P., Acevedo-Arozena A. (2021). Guidelines for the use and interpretation of assays for monitoring autophagy (4th edition). Autophagy.

[29] Stoddart M.J. (2011). Cell viability assays: Introduction. Methods Mol. Biol..

[30] Schenborn E.T. (2000). Transfection technologies. Methods Mol. Biol..

[31] Bong D., Sohn J., Lee S.V. (2024). Brief guide to RT-qPCR. Mol. Cells.

[32] Qu H., Yuan X., Huang K., Liu D. (2025). AKT/mTOR mediated autophagy contributes to the self-replication of canine influenza virus in vivo and in vitro. Cell Signal.

[33] Yan L.J., Allen D.C. (2021). Cadmium-Induced Kidney Injury: Oxidative Damage as a Unifying Mechanism. Biomolecules.

[34] Yuan W., Liang L., Huang K., Deng Y., Dong M., Wang G., Zou F. (2020). MiR-122-5p and miR-326-3p promote cadmium-induced NRK-52E cell apoptosis by downregulating PLD1. Environ. Toxicol..

[35] Rahman Z., Singh V.P. (2019). The relative impact of toxic heavy metals (THMs) (arsenic (As), cadmium (Cd), chromium (Cr)(VI), mercury (Hg), and lead (Pb)) on the total environment: An overview. Environ. Monit. Assess..

[36] Zhou J., Huang Y., Li G., Zhao Z., Deng Y., Xian S., Hu Y., Yi M., Liu L. (2026). Cadmium exposure induces renal fibrosis by inhibiting hsa_circ_0075684/miR-363-3p/KLF4 signaling pathway. Sci. Rep..

[37] Qiu W., Ye J., Su Y., Zhang X., Pang X., Liao J., Wang R., Zhao C., Zhang H., Hu L. (2023). Co-exposure to environmentally relevant concentrations of cadmium and polystyrene nanoplastics induced oxidative stress, ferroptosis and excessive mitophagy in mice kidney. Environ. Pollut..

[38] Ding L., Wang K., Zhu H., Liu Z., Wang J. (2024). Protective effect of quercetin on cadmium-induced kidney apoptosis in rats based on PERK signaling pathway. J. Trace Elem. Med. Biol..

[39] Fitton J.H., Park A.Y., Karpiniec S.S., Stringer D.N. (2020). Fucoidan and Lung Function: Value in Viral Infection. Mar. Drugs.

[40] Dimitrova-Shumkovska J., Krstanoski L., Veenman L. (2020). Potential Beneficial Actions of Fucoidan in Brain and Liver Injury, Disease, and Intoxication-Potential Implication of Sirtuins. Mar. Drugs.

[41] Lean Q.Y., Eri R.D., Fitton J.H., Patel R.P., Gueven N. (2015). Fucoidan Extracts Ameliorate Acute Colitis. PLoS ONE.

[42] Jayasinghe A.M.K., Kirindage K., Fernando I.P.S., Han E.J., Oh G.W., Jung W.K., Ahn G. (2022). Fucoidan Isolated from *Sargassum confusum* Suppresses Inflammatory Responses and Oxidative Stress in TNF-α/IFN-γ- Stimulated HaCaT Keratinocytes by Activating Nrf2/HO-1 Signaling Pathway. Mar. Drugs.

[43] Huwait E., Al-Saedi D.A., Mirza Z. (2022). Anti-Inflammatory Potential of Fucoidan for Atherosclerosis: In Silico and In Vitro Studies in THP-1 Cells. Molecules.

[44] Kim E.A., Lee S.H., Ko C.I., Cha S.H., Kang M.C., Kang S.M., Ko S.C., Lee W.W., Ko J.Y., Lee J.H. (2014). Protective effect of fucoidan against AAPH-induced oxidative stress in zebrafish model. Carbohydr. Polym..

[45] Livingston M.J., Zhang M., Kwon S.H., Chen J.K., Li H., Manicassamy S., Dong Z. (2024). Autophagy activates EGR1 via MAPK/ERK to induce FGF2 in renal tubular cells for fibroblast activation and fibrosis during maladaptive kidney repair. Autophagy.

[46] Minami S., Yamamoto T., Yamamoto-Imoto H., Isaka Y., Hamasaki M. (2023). Autophagy and kidney aging. Prog. Biophys. Mol. Biol..

[47] Dong P.F., Liu T.B., Chen K., Li D., Li Y., Lian C.Y., Wang Z.Y., Wang L. (2025). Cadmium targeting transcription factor EB to inhibit autophagy-lysosome function contributes to acute kidney injury. J. Adv. Res..

[48] Ma Y., Yue C., Sun Q., Wang Y., Gong Z., Zhang K., Da J., Zou H., Zhu J., Zhao H. (2023). Cadmium exposure exacerbates kidney damage by inhibiting autophagy in diabetic rats. Ecotoxicol. Environ. Saf..

[49] Hu Y., Wang K., Xu J., Wan G., Zhao Y., Chen Y., Jiang K., Li X. (2025). mTOR-Mediated Autophagy Regulates Cadmium-Induced Kidney Injury via Pyroptosis. Int. J. Mol. Sci..

[50] Chen M., Wang J., Zhang P., Jiang Z., Chen S., Liang S., Ma T., Liao H., Tan W., Niu C. (2025). Low molecular weight fucoidan induces M2 macrophage polarization to attenuate inflammation through activation of the AMPK/mTOR autophagy pathway. Eur. J. Pharmacol..

[51] Settembre C., Di Malta C., Polito V.A., Garcia Arencibia M., Vetrini F., Erdin S., Erdin S.U., Huynh T., Medina D., Colella P. (2011). TFEB links autophagy to lysosomal biogenesis. Science.

[52] Martini-Stoica H., Xu Y., Ballabio A., Zheng H. (2016). The Autophagy-Lysosomal Pathway in Neurodegeneration: A TFEB Perspective. Trends Neurosci..

[53] Pi H., Li M., Zou L., Yang M., Deng P., Fan T., Liu M., Tian L., Tu M., Xie J. (2019). AKT inhibition-mediated dephosphorylation of TFE3 promotes overactive autophagy independent of MTORC1 in cadmium-exposed bone mesenchymal stem cells. Autophagy.

[54] Wang T., Yan L., Wang L., Sun J., Qu H., Ma Y., Song R., Tong X., Zhu J., Yuan Y. (2023). VPS41-mediated incomplete autophagy aggravates cadmium-induced apoptosis in mouse hepatocytes. J. Hazard. Mater..

[55] Zhao J., Hu B., Xiao H., Yang Q., Cao Q., Li X., Zhang Q., Ji A., Song S. (2021). Fucoidan reduces lipid accumulation by promoting foam cell autophagy via TFEB. Carbohydr. Polym..

[56] Zhang W., Li X., Wang S., Chen Y., Liu H. (2020). Regulation of TFEB activity and its potential as a therapeutic target against kidney diseases. Cell Death Discov..

[57] Cui Z., Napolitano G., de Araujo M.E.G., Esposito A., Monfregola J., Huber L.A., Ballabio A., Hurley J.H. (2023). Structure of the lysosomal mTORC1-TFEB-Rag-Ragulator megacomplex. Nature.

[58] Tan A., Prasad R., Lee C., Jho E.H. (2022). Past, present, and future perspectives of transcription factor EB (TFEB): Mechanisms of regulation and association with disease. Cell Death Differ..

