# Supplementary material for: Fucoidan Protects Against Cadmium-Induced Cytotoxicity in PK-15 Cells by Restoring Autophagic Flux: Involvement of the TFEB Signaling Pathway
Source: Toxics. 2026 May 13;14(5):430. doi: 10.3390/toxics14050430 (PMC13211490; doi:10.3390/toxics14050430)
Supplement: Supplementary file 1 [file toxics-14-00430-s001.zip › toxics-4242920-supplementary.pdf]

Supporting Information for Publication

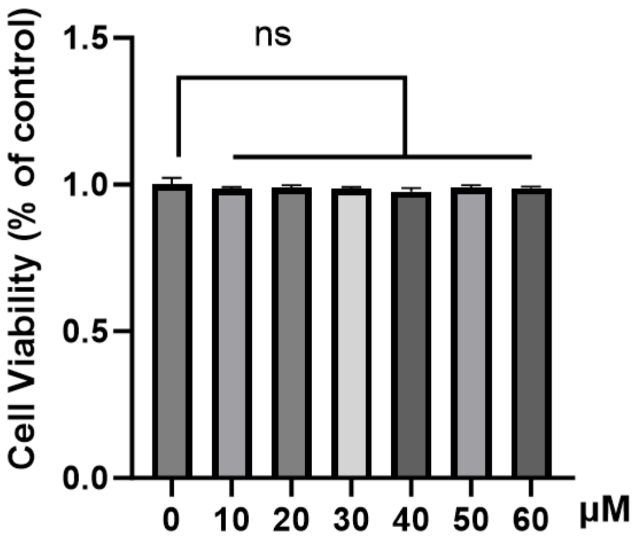

Figure S1. Effect of different concentrations of chloroquine (CQ) on cell viability after 4 h treatment. Cells were treated with CQ at concentrations of 0, 10, 20, 30, 40, 50, and 60 μM for 4 h, and cell viability was measured by CCK-8 assay and expressed as a percentage of the control (0 μM) group. Data are presented as mean ± SD (n=3). ns, not significant (one-way ANOVA followed by Tukey's post hoc test).

Table S1 The primer sequences for each gene

Gene	Species	Primer sequence (5'-3')

GAPDH	Swine	F: GTCGGAGTGAACGGATTTGGC<br>R: GGAGGTCAATGAAGGGGTCA
IL-1β	Swine	F: GCCAGTCTTCATTGTTCAGGTTT<br>R: ATCTCTTTGGGGCCATCAGC
TNF-α	Swine	F: GCCCAAGGACTCAGATCATCG<br>R: GGCATACCCACTCTGCCATT
NGAL	Swine	F: CTATGTCGTGCGTGTGGTGTCTAC<br>R: TCCTCCCGTAAAGCGTGGTTTG
Ntn-1	Swine	F: TCCTCACCGATCTCAACAACCC<br>R: CTCACGTAGGTCACCTCGAAC
Kim-1	Swine	F: ACTGTCCATACAGGTGCTGC<br>R: TGAGCAAGAGGGCATCAGTG
ATG5	Swine	F: GTGCTTCGAGATGTGTGGTT<br>R: GTGCTTCGAGATGTGTGGTT
ATG10	Swine	F: TGCTATAAGACACGGCTGCT<br>R:TGAGCAGAAGGGAAGAATCACAT
P62	Swine	F: CTGAGACATGGGCACTTCGG<br>R: TGATGGACCAGAAGCTGATTCC
LC3B	Swine	F: ACGAAATTCCTGGTGCCTGA<br>R: AGCTGCAAGCGCCTTCTAAT
